# Occult small bowel perforation in a patient with Ehlers Danlos syndrome: a case report and review of the literature

**DOI:** 10.1186/1757-1626-3-57

**Published:** 2010-02-12

**Authors:** Tessa Frances Leake, Tarun Singhal, Aninda Chandra, Alexandra Ashcroft, Sudeendra Doddi, Abdulzahra Hussain, Frank Smedley

**Affiliations:** 1 Department of General and Colorectal Surgery, Princess Royal University Hospital, Farnborough Common, Orpington, Greater London, Kent, BR6 8ND, UK

## Abstract

Patients who present with a co-existing connective tissue disorder add a degree of complexity to operative intervention. We present an unusual case of a 53-year-old Caucasian female patient with Ehlers Danlos syndrome who presented with an occult perforation of the distal ileum. The patient had known small bowel diverticulae yet the perforation occurred within the normal bowel wall. The pre-operative CT only showed malrotation of the large bowel and did not correlate with the intra-operative findings. Our case has highlighted that although small bowel perforation is a rare occurrence, it may be more common in Ehlers Danlos and may present with atypical features. Perforation may also occur alongside normal bowel as well as diverticulae within the bowel. Where diverticulae exists within a patient with Ehlers Danlos syndrome and there is some diagnostic uncertainty, there should be a lower threshold for operative intervention. We present in the discussion a number of salient features and learning points.

## Introduction

Patients who present with a co-existing connective tissue disorder add a degree of complexity to operative intervention. Ehlers Danlos syndrome is a group of disorders that affect connective tissue and in type IV leads to the absence of type III collagen in the bowel wall [1]. This can result in a variety of surgical emergency presentations. We present an unusual case of a 53-year-old female Caucasian with Ehlers Danlos syndrome who presented with an occult perforation of the distal ileum within normal bowel wall on a background of multiple small bowel diverticulae. Radiographic investigation did not demonstrate the cause of the patient's clinical deterioration. A subsequent laparotomy revealed multiple small bowel diverticulae and perforations. In light of the operative findings, we reviewed the literature pertaining to Ehlers Danlos, small bowel diverticulae and perforation. We present in the discussion a number of salient features and learning points.

## Case presentation

**Figure 1:**
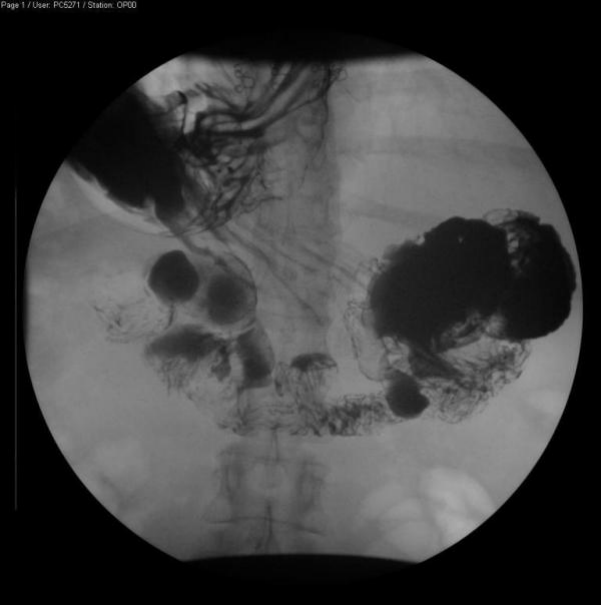
**This is a gastrograffin meal highlighting diverticulae in the duodenum, performed six weeks prior to admission for further evaluation of oesophagitis**.

A 53-year-old Caucasian female with Ehlers Danlos syndrome presented with abdominal pain and vomiting to the Emergency Medicine Department. The pain was described as aching in character, initially beginning in the left iliac fossa and radiating to the left upper quadrant. The patient was unable to keep food down and had several episodes of vomiting over the course of the previous 24 hours. She felt distended but the bowel motion was unchanged with normal stools on the day of admission and no rectal bleeding. The patient recounted feeling feverish prior to admission. She had a previous history of diverticular disease and had seen her general practitioner (GP) four days earlier with abdominal pain. Her GP had started her empirically on oral Cefalexin and Metronidazole. Her past medical history consisted of diverticulosis within both the small and large bowel diagnosed on contrast studies (Figure 1). She also had oesophagitis. The patient had also had two previous caesarean section deliveries which were uncomplicated and a right-sided inguinal hernia repair. The patient was neither a smoker nor drinker. The only history relating to her Ehlers Danlos was of thin skin and an increased bruising tendency, with mild difficulty in using her hands since childhood with occasional finger locking and decreased hand strength. There was no significant family history and none of connective tissue disorders.

On examination the abdomen was soft and minimally tender with no guarding, rigidity or rebound tenderness. Bowel sounds were present. Rectal examination revealed soft stool in the rectum. Chest radiograph was normal and abdominal films showed distended bowel loops. The patient's bloods on admission showed a normal white cell count of 6.9 and a raised C - reactive protein of 491. The other bloods were normal. The surgical team organised admission and converted her to intravenous antibiotics and fluids.

**Figure 2:**
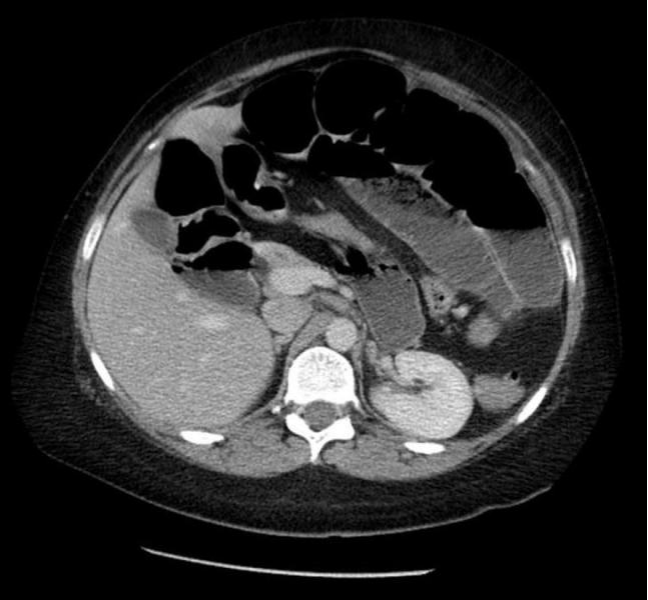
**This is a CT Scan showing malrotated large loopy colon and distended small bowel, taken one day before operation performed on the patient**.

Over the next 48 hours, the abdominal pain continued to increase and was associated with intermittent vomiting with an inability to eat or drink. C - reactive protein remained elevated. The patient continued to feel distended yet bowels continued to open normally. A repeat abdominal radiograph revealed progressive bowel distension so a CT scan was requested. This was performed the same day and suggested malrotation of the large bowel with a dilated caecum lying under the diaphragm on the left hand side and the ascending colon lying to the left of the midline (Figure 2). Multiple loops of dilated jejunum and ileum were present. Engorged vessels were present in the root of the mesentery and tortuosity of the mesenteric vessels was also noted. There was no ascites or free intraperitoneal air and no obvious ischaemia of the bowel.

**Figure 3:**
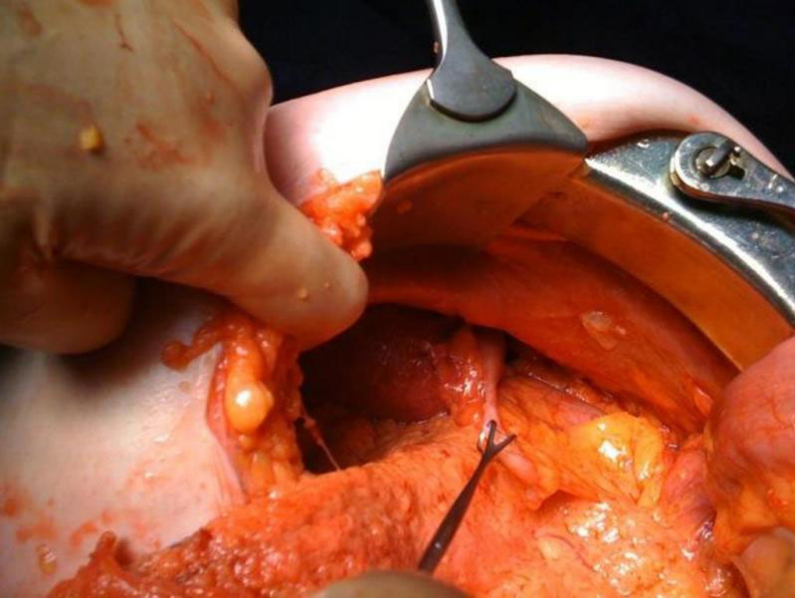
**This is an intra-operative image showing malrotation with the caecum fixed to the left hypochondrium**.

**Figure 4:**
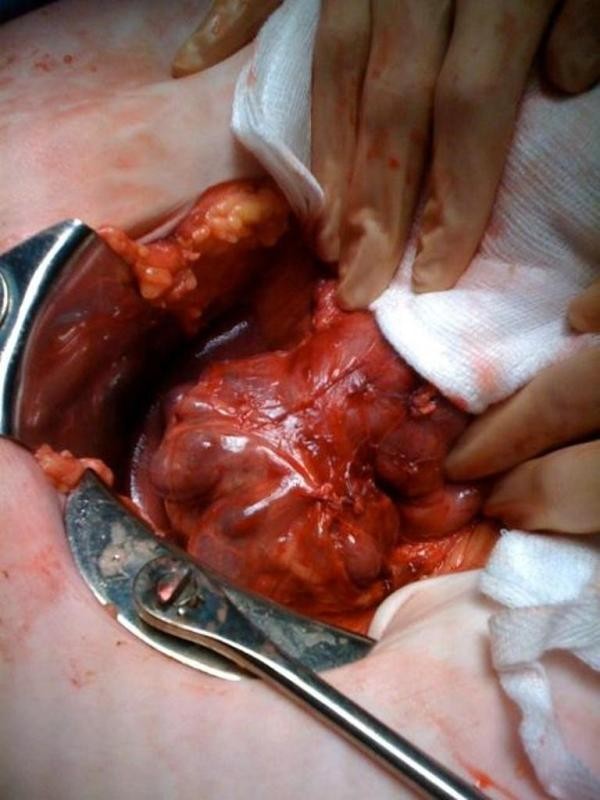
**This is an intra-operative image showing large, thin walled, diverticulae in the duodenum with bowel contents visible through the wall of the diverticlae**.

**Figure 5:**
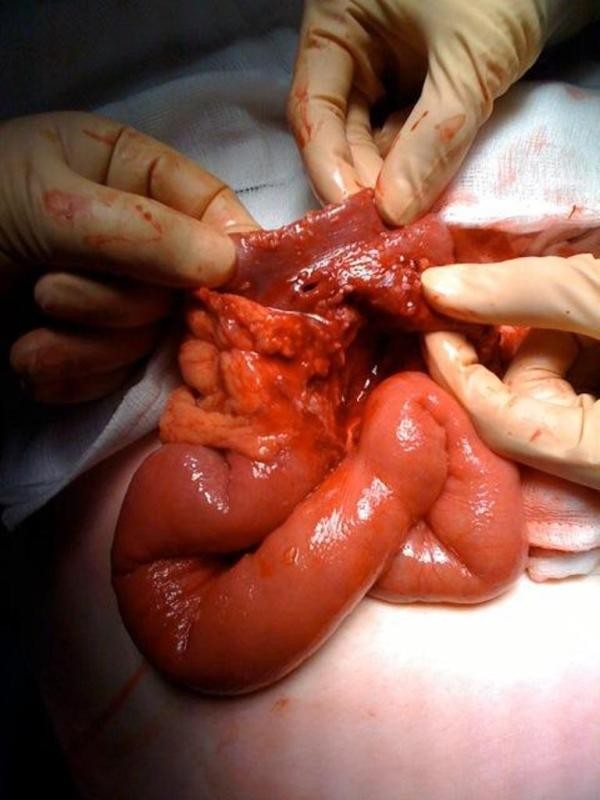
**This is an intra-operative image showing the perforations were in the segment of distal ileum with no diverticulae**.

**Figure 6:**
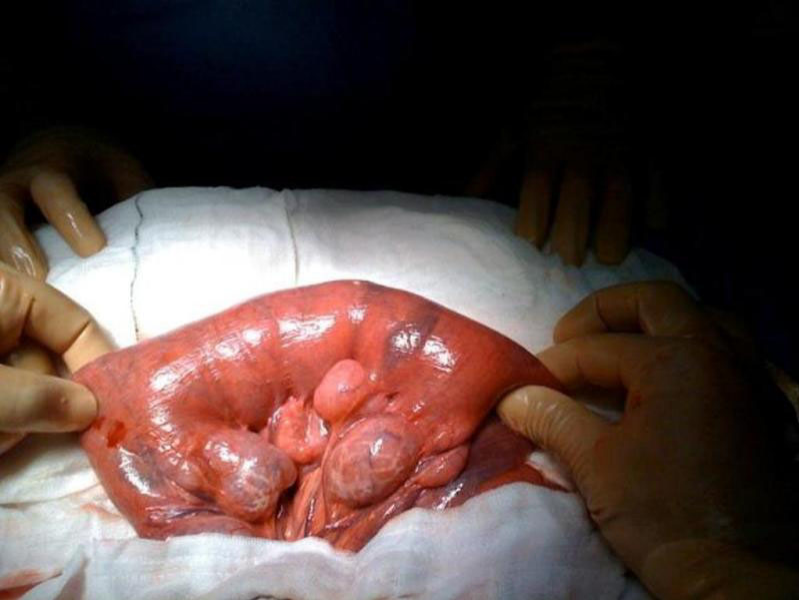
**This is an intra-operative image showing proximal ileum and jejunum showing large, thin walled diverticulae, which were fluid filled**.

In view of the continuing deterioration in conjunction with the imaging, a laparotomy was performed. Malrotation of the bowel was found as per the CT findings and the caecum was fixed in the left hypochondrium (Figure 3). Numerous diverticulae in the duodenum were also noted (Figure 4). Multiple perforations sealed with omentum were seen within a six-inch segment of distal ileum without perforations of diverticulae as such. The perforations occurred within relatively normal bowel (Figure 5). There was massive dilatation of the small intestine with huge multiple diverticulae especially in the jejunum and proximal ileum (Figure 6). Adhesions were then divided with sharp dissection and the bowel wall was noted to be extremely fragile. Serosal tears were evident with minimal handling (Figure 4).

The small intestine segment with the multiple perforations was resected. A hand sutured double-layer anastomosis was performed. The mesenteric defect was also closed. Haemostasis was obtained and an abdominal lavage performed with two litres of warmed normal saline. A corrugated drain was brought out through the right iliac fossa. Mass closure was performed with loop 0 Nylon. Wound lavage was performed and clips used to appose skin.

Post-operatively, intravenous antibiotics were prescribed, and the patient was admitted to the intensive care unit. Total parenteral nutrition was started and the patient kept nil by mouth. Her bloods showed a decreasing CRP from 175 to 32 and her WCC remained stable. The patient's haemoglobin dropped to 6.0 gm/dl post-operatively while her platelets climbed to 1532. The patient refused a blood transfusion. Also during the post-operative period there was a developing mild reduction in peripheral oxygenation noted incidentally with accompanying blue hands and lips.

The patient made a good post-operative recovery with discharge to the ward on day 4 with commencement of a normal diet by post-operative day 7. Resumption of normal bowel habit began and patient was discharged home. Follow-up over the next three months confirmed no other complications.

Histology revealed a segment of small intestine measuring 16 cm in length × 3 cm in diameter. The serosal surface was noted to be haemorrhagic, with several perforations, the largest measuring 1.0 × 0.5 cm with associated white exudate on the serosa. The mucosa shows a submucosal haemorrhage measuring 1.0 × 1.0 cm, which was 4 cm from its closest margin. The second specimen revealed an omental mass with an appearance of white tissue measuring 2.5 × 1.5 × 1.0 cm.

On microscopy, the sections of small bowel show submucosal oedema and vascular dilatation with focal haemorrhage and perforation and organising inflammation in the serosal surface. The mucosa was preserved and showed no significant abnormality. The resection margins showed viable small bowel. There was no dysplasia or malignancy and there was no thrombus in the sections of the mesenteric vessels.

Further sections of the omental mass show organising inflammatory debris and granulation tissue. No malignant cells were seen. Within the small bowel resection a submucosal haemorrhage and focal perforation was noted. On microscopy of the omental mass, inflammatory debris was seen.

## Discussion

Ehlers Danlos syndrome type IV is an often lethal disease caused by various mutations of type III collagen genes [2]. Affected individuals have fragile connective tissue, bruise easily and are loose-jointed. Seven categories can be derived on clinical, genetic, and biochemical grounds [3]. In type IV Ehlers Danlos syndrome, the patient's skin is tight and thin with visible veins to the naked eye, particularly over the face and ears. Some individuals paradoxically display a tendency to form keloids and contractures despite the deficiency of collagen [3].

In Ehlers Danlos syndrome type IV, one of the genetically distinct collagens (type III) normally found in skin, aorta, and intestine is missing from the tissues of patients [2] and only type I collagen is synthesised. The gene for human type III collagen has been cloned firstly as a cDNA covering the 3' end, 5 and more recently as full genomic sequences [1]. It is a group of heterogenous disorders either presenting as a new mutation or as part of a familial pattern. Ehlers Danlos syndrome type IV has two main phenotypes with the 'acrogeric' form having a much worse prognosis than the 'ecchymotic' form. Our case was likely the ecchymotic form type IV. The acrogeric phenotype tends to have large eyes, peaked nose and premature ageing of the hands [1]. This phenotype includes the more serious and lethal sequelae of vascular rupture of the aorta in later life. It can present in many ways but the commonest is with gastrointestinal (GI) side effects. The two main complications include GI bleeding and perforation. Of the perforations, most of these occur within the colon, more specifically the recto-sigmoid junction. A study performed by Sigurdson et al revealed that in-vitro electromyographic studies of the colonic tissue suggested a possible link between abnormal myogenic activity and colonic perforations [4]. Less commonly the small bowel can perforate both within diverticulae and within normal bowel as with our case. A case report by Aldridge in 1967 highlighted perforations in the normal bowel as well as that of the diverticulae [5]. Alimentary bleeding preceded the perforation in several of these individuals, and in view of the changes which were disclosed at operation it seems possible that bleeding into the wall of the gut might precede local necrosis and subsequent perforation [5].

In a case series of 125 patients reported by Beighton et al [3], two developed perforation, one of the colon and another of the jejunum. Several other patients reported recurrent, vague, ill-defined abdominal pains. These had been thought to represent episodes of partial obstruction due to intussusception or volvulus. Various case reports of tissue friability include rectal bleeding on passing of hard motions. Several patients in the survey reported rectal bleeding following the passage of hard stools [3]. Tissue friability is also noted by many operating surgeons on patients with Ehlers Danlos syndrome due to the collagen deficiency. With many intestinal operations serosal tears occur on minimal handling and so a minimally invasive/minimal handling approach is advised when working with the bowel.

In our case, the patient developed perforations, associated with symptoms of mild gastritis and had no episode of GI bleed. In the literature, a permanent stoma is recommended as the operative approach due to the high incidence of anastomotic leak. In a review in Japanese literature, when colonic perforation occurs in this syndrome, total colectomy and ileo-rectal anastomosis is reasonably indicated [6]. The recommendation is either an end colostomy or Hartmann's procedure. The reported experience although small, indicates that a high incidence of recurrent perforation can be expected if bowel continuity is re-established [7]. A decision was made not to leave a stoma as the ends for anastomosis were free of diverticulae and suitable for anastomosis. In our case the bowel was hand sutured and a primary anastomosis was achieved. The bowel was friable and the wall was oedematous therefore making stapled anastomosis unreliable and hand suture technique more favourable.

## Conclusion

Our case has highlighted that although GI perforations can occur in Ehlers Danlos it can be a difficult initial diagnosis, and small bowel perforation is a rare occurrence. Perforation may also occur alongside normal bowel as well as diverticulae within the bowel. It is also important to realise that diverticulae can spread from the colon to the stomach and so symptoms of perforation may be varied or atypical. Where jejunal diverticulae exists and there is some diagnostic uncertainty, there should be a lower threshold for operative intervention. It is also important to highlight the significance of minimal bowel handling and to be aware of the complications of primary anastomosis in patients with Ehlers Danlos Syndrome.

## Abbreviations

ED: Ehlers Danlos

## Consent

Written informed consent was obtained from the patient for publication of this case report and accompanying images. A copy of the written consent is available for review by the Editor-in-Chief of this journal.

## Competing interests

The authors declare that they have no competing interests.

## Authors' contributions

TL and AC performed literature search and prepared case report, TS, SD, AH, FS contributed to case report and discussion while AA helped in reviewing the literature. All authors read and approved the final manuscript.
